# Characterization of the cellular transport mechanisms for the anti‐cachexia candidate compound TCMCB07

**DOI:** 10.1002/jcsm.12602

**Published:** 2020-07-29

**Authors:** Yongjun Hu, Kenneth A. Gruber, David E. Smith

**Affiliations:** ^1^ Department of Pharmaceutical Sciences, College of Pharmacy University of Michigan Ann Arbor MI USA; ^2^ Tensive Controls, Inc. Columbia MO USA

**Keywords:** ABC transporters, Cachexia, Mass balance, Melanocortins, SLC transporters, TCMCB07

## Abstract

**Background:**

Cachexia is a debilitating, life‐threatening condition whose pathology includes reduced food intake accompanied by hypermetabolism, leading to a catabolic state. The hypothalamic melanocortin system is a critical regulator of metabolic rate with effects being mediated through the melanocortin‐4 receptor (MC4R). MC4R activation is also critical to the initiation and maintenance of cachexia. A major problem in the design of anti‐cachexia drugs has been the need to cross the blood–brain barrier to access the metabolic rate‐controlling centres in the hypothalamus. The overwhelming majority of anti‐cachexia drugs are only effective when administered intracerebroventricularly. TCMCB07 is a cyclic nonapeptide peptide MC4R antagonist with parenteral anti‐cachexia activity in both small and large animal models. This suggests it can cross the blood–brain barrier. The aim of this study was to examine potential transport mechanisms of TCMCB07 furthering its preclinical development for subsequent studies in humans.

**Methods:**

*In vitro* studies were performed in transporter‐transfected cells to study whether or not TCMCB07 was an inhibitor as well as substrate for OATP1A2, OATP1B1, OATP1B3, OATP2B1, OCT2, OAT1, OAT3, MATE1, MATE2‐K, P‐gp (MDR1), and BCRP. *In vivo* mass balance studies were also performed in mice to evaluate the absorption and disposition of TCMCB07 after oral and intravenous bolus administrations.

**Results:**

TCMCB07 inhibited the uptake of prototypical substrates in cells transfected with OATP1A2 (IC_50_ 24.0 μM), OATP1B1 (IC_50_ 6.8 μM), OATP1B3 (IC_50_ 307 μM), OATP2B1 (IC_50_ 524 μM), OCT2 (IC_50_ 1,169 μM), MATE1 (IC_50_ 8.7 μM), and MATE2‐K (IC_50_ 20.7 μM) but not in cells transfected with OAT1 and OAT3. TCMCB07 did not affect the P‐gp (MDR1)‐mediated and BCRP‐mediated permeability of prototypical substrates in transfected cells. Importantly, direct evidence was shown for the uptake of TCMCB07 in OATP1A2‐transfected cells (i.e. *V*_max_ 236 pmol/mg, *K*_*m*_ 58.4 μM, and *K*_*d*_ 0.39 μL/mg), demonstrating that the nonapeptide was a substrate for this transporter. Mass balance studies demonstrated that 24.2% of TCMCB07 was absorbed orally *in vivo* (*P* = 0.0033) and excreted primarily in the bile after both oral and intravenous administrations.

**Conclusions:**

OATP1A2 is the transporter responsible for the oral absorption of TCMCB07 in the intestine and for its pharmacologic response in the brain.

## Introduction

Cachexia is a muscle wasting disorder, with or without loss of fat mass, in which a person suffers from a chronic illness such as congestive heart failure, chronic obstructive pulmonary disease, chronic kidney disease, acquired immunodeficiency syndrome, and cancer, and there is a loss of more than 5% body weight in ≤12 months.^1^, ^2^, ^3^ Cachexia also results in endocrine, metabolic, and central nervous system disturbances, a reduced quality of life, and increased mortality. Importantly, cachexia is distinct from starvation and simple malnutrition in which the latter two can be reversed by the provision of adequate nourishment. At present, there are no approved drugs that completely reverse cachexia, and as a result, this malady remains a major unmet medical need.

Several pharmacological strategies have been examined for the treatment of cachexia in general and for the treatment of cancer‐related cachexia in particular.^1^, ^2^, ^4^, ^5^ These strategies have included compounds for appetite stimulation [e.g. megestrol acetate, ghrelin agonists, and melanocortin‐4 receptor (MC4R) antagonists], anti‐inflammatory agents (e.g. cyclooxygenase‐2 inhibitors, thalidomide, and anti‐interleukin‐1 and anti‐interleukin‐6 monoclonal antibodies), selective androgen receptor modulators (e.g. enobosarm and LGD‐4033), and others. More recently, a novel MC4R antagonist, TCMCB07, has been reported in preliminary studies to show efficacy and safety in rat renal and cancer‐induced cachexia,^6^ as well as in normal and companion dogs with cachexia.^7^ It was further reported that TCMCB07 had oral anti‐cachexia activity, implying both intestinal and blood–brain barrier (BBB) transport.^7^ The same authors also showed hepatic uptake of TCMCB07 and secretion into bile, implying the involvement of hepatic transporters.

On the basis of the localization of drug transporters in the intestine, liver, and brain,^8^ and what we know about the absorption and disposition of TCMCB07, we hypothesized that the most likely candidate transporter(s) of TCMCB07 were organic anion‐transporting polypeptides (OATPs). Thus, using *in vitro* studies in transfected cell cultures, our primary aim was to determine the transport mechanisms responsible for the oral absorption, hepatic/biliary secretion, and BBB uptake of TCMCB07. Our secondary aim, using mass balance studies in mice, was to confirm the *in vivo* oral absorption and excretion pattern of TCMCB07.

## Methods

### Materials

**Figure 1:**
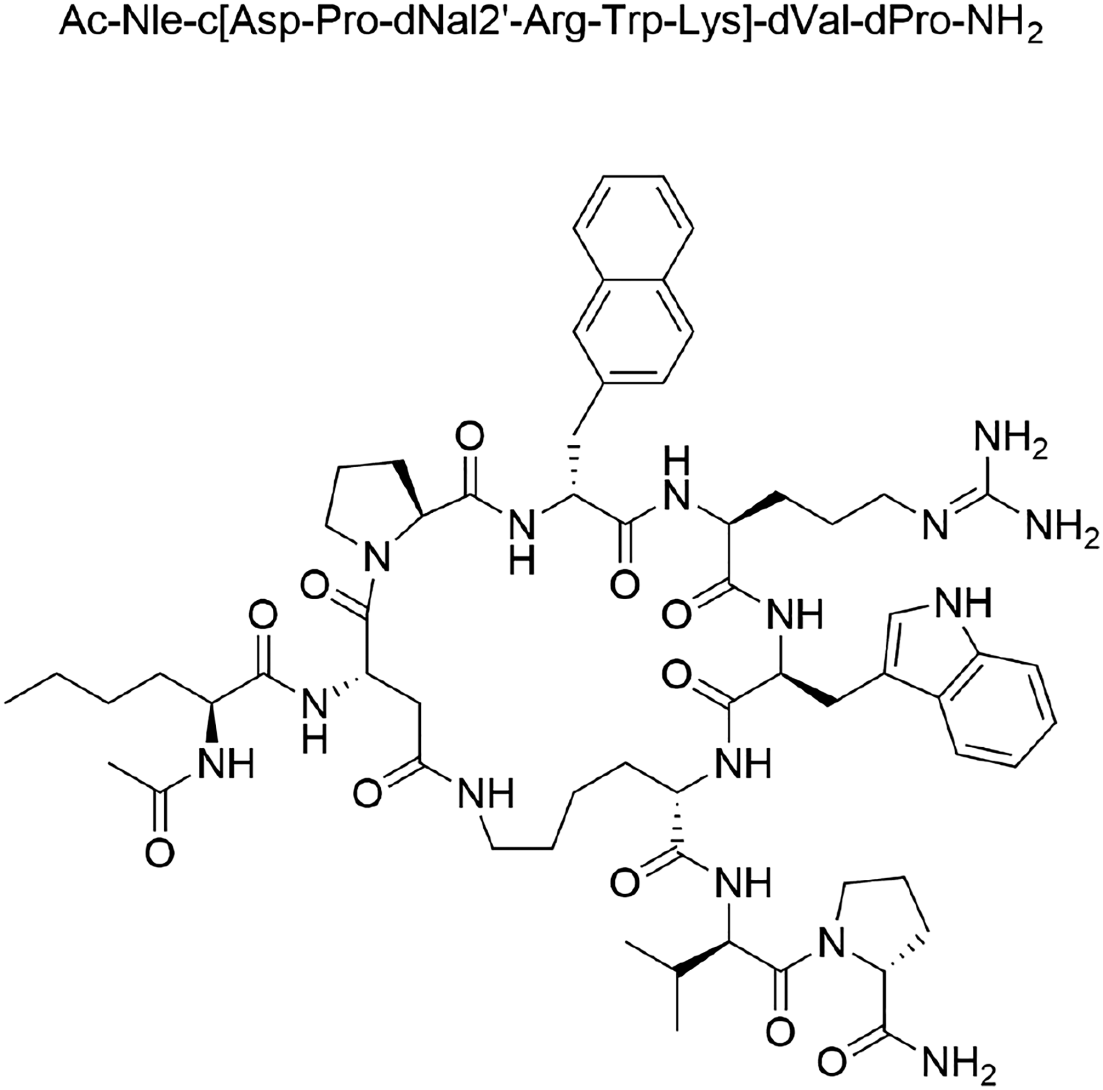
Chemical structure of TCMCB07.

TCMCB07 (Ac‐Nle‐c[Asp‐Pro‐dNal2′‐Arg‐Trp‐Lys]‐dVal‐dPro‐NH_2_), a cyclic nonapeptide (*Figure* *1*), was provided by Tensive Controls, Inc. (Columbia, MO). Radiolabelled TCMCB07 (1‐^14^C‐Ac‐Nle‐c[Asp‐Pro‐dNal2′‐Arg‐Trp‐Lys]‐dVal‐dPro‐NH_2_; 50 mCi/mmol; 98.6% purity) was custom synthesized by Polypeptide Laboratories (San Diego, CA). HEK293 cell cultures transfected with the human clones OATP1B1, OATP1B3, and OATP2B1 were a generous gift of Bristol‐Myers Squibb, Lawrenceville, NJ; HEK293 cell cultures transfected with the human clone OATP1A2 was a generous gift of Dr Markus Keiser (University of Greifswald, Germany); HEK293 cell cultures transfected with the human clones OAT1, OAT3, OCT2, MATE1, and MATE2‐K were a generous gift of Dr Kathleen Giacomini (University of California, San Francisco); and MDCKII cell cultures transfected with the human clones MDR1 and BCRP were provided by Dr Alfred Schinkel (Netherlands Cancer Institute, Amsterdam). Radiolabelled substrates purchased from PerkinElmer (Waltham, MA) included [^3^H]estradiol‐17‐β‐d‐glucuronide (52.9 Ci/mmol) for OATP1B1, [^3^H]cholecystokinin octapeptide (83 Ci/mmol) for OATP1B3, [^3^H]estrone 3‐sulfate (51.8 Ci/mmol) for OATP2B1, OATP1A2, and OAT3, [^14^C]p‐aminohippurate (52.7 mCi/mmol) for OAT1, [^3^H]1‐methyl‐4‐phenylpyridinium (79.9 Ci/mmol) for OCT2, [^14^C]tetraethylammonium (3.5 mCi/mmol) for MATE1 and MATE2‐K, and [^3^H]digoxin (26.3 Ci/mmol) for MDR1. [^3^H]Rosuvastatin (11.7 Ci/mmol) for BCRP was purchased from Moravek, Inc. (Brea, CA). [^3^H]Mannitol (15.8 Ci/mmol) and [^14^C]mannitol (57 mCi/mmol) were also purchased from PerkinElmer. All other chemicals were obtained from standard sources.

### Cell culture studies in HEK293/OATP1B1, HEK293/OATP1B3, HEK293/OATP2B1, HEK293/OAT1, HEK293/OAT3, and HEK293/OCT2

Mock and transfected cells (500 000 cells/well) were seeded onto BioCoat poly‐d‐lysine 24 well plates (Corning Inc., Corning, NY) and cultured in Dulbecco's modified Eagle's media (high glucose, high glutamine, sodium pyruvate, 10% fetal bovine serum, and 0.1 mM minimum essential medium non‐essential amino acid solution), 37°C and humidified 95% CO_2_, for 2 to 4 days as described previously.^9^, ^10^, ^11^ Media were changed every 24 h. Cells were then washed three times × 1.0 mL each with pre‐warmed Hank's Balanced Salt Solution (HBSS) buffer. A 0.5 mL aliquot of uptake buffer [HBSS plus 10 mM 4‐(2‐hydroxyethyl)‐1‐piperazineethanesulfonic acid (HEPES), pH 7.4], containing the radiolabelled prototypical substrate and mannitol, was then incubated for various times up to 60 min to determine the optimal incubation time for subsequent experiments (i.e. time of sampling during linear uptake conditions). At the designated times, 1.0 mL of ice‐cold HBSS buffer was added to stop uptake after which the cells were rinsed three times with 1.0 mL with ice‐cold HBSS. Hyamine hydroxide (0.33 mL) was then added to each well to lyse the cells. The cells were transferred into a scintillation vial and mixed with 6.0 mL of cytosine scintillation cocktail. Radioactivity of the solubilized cells was determined with a dual‐channel liquid scintillation counter (Beckman LS 6000; Beckman Coulter Inc., Fullerton, CA) and the protein quantified by a bicinchoninic acid protein assay kit (Pierce Biotechnology; Rockford, IL). For dose–response studies and the estimation of IC_50_ values, the uptake buffer also contained unlabelled TCMCB07 over the 0.1–1000 μM concentration range.

### Cell cultures studies in HEK293/OATP1A2

Mock and transfected cells (250 000 cells/well) were seeded onto BioCoat poly‐d‐lysine 24 well plates (Corning Inc.) and cultured for 2 days as described previously for the other OATPs. However, for these studies, the cells were pretreated for 24 h with 5 mM sodium butyrate in order to increase transporter expression.^12^ A different uptake buffer was also used (142 mM NaCl, 5 mM KCl, 1 mM K_2_HPO_4_, 1.2 mM MgSO_4_, 1.5 mM CaCl_2_, 5 mM glucose, and 12.5 mM HEPES, pH 7.3) along with ice‐cold phosphate‐buffered saline (pH 7.4) to stop uptake. Cells were incubated for various times over 10 min for the uptake studies. Radioactivity and protein measurements were then performed as described previously. For concentration‐dependent studies, the 5 min uptake of [^14^C]TCMCB07 was evaluated over total drug concentrations of 2–500 μM.

### Cell culture studies in HEK293/MATE1 and HEK293/MATE2‐K

Mock and transfected cells (250 000 cells/well) were seeded onto BioCoat poly‐d‐lysine 24 well plates (Corning Inc.) and cultured for 2 days as described previously. However, for these studies, a different incubation buffer was used (145 mM NaCl, 3 mM KCl, 1 mM CaCl_2_, 0.5 mM MgCl_2_, 5 mM glucose, and 5 mM HEPES, pH 7.4).^13^ Moreover, intracellular acidification was achieved by preincubating the cells in buffer containing 30 mM ammonium chloride for 20 min at 37°C. Cells were then incubated for various times over 10 min for the uptake studies. Radioactivity and protein measurements were performed as described previously.

### Cell culture studies in MDCKII/MDR1 and MDCKII/BCRP

Mock and transfected cells (40 000 cells/well) were seeded onto laminin‐coated Falcon 24 well multiwell inserts (Corning Inc.) and cultured in Dulbecco's modified Eagle's media (high glucose, high glutamine, sodium pyruvate, 10% fetal bovine serum, and 0.1 mM minimum essential medium non‐essential amino acid solution), 37°C and humidified 95% CO_2_, for 2 days as described previously.^14^, ^15^ Media were changed every 24 h. Cells were then washed three times, each with 0.5 mL pre‐warmed HBSS buffer. For transepithelial transport in the A to B direction, 0.3 mL of drug‐containing buffer pH 7.4 (HBSS, 25 mM HEPES, 0.5% DMSO, 38 nM [^3^H]digoxin or 21 nM [^3^H]rosuvastatin, and 1.75 μM [^14^C]mannitol) was added to the apical chamber, and 1.0 mL of blank buffer (i.e. no drug) was added to the basolateral chamber. For transepithelial transport in the B to A direction, 1.0 mL of drug‐containing buffer pH 7.4 (HBSS, 25 mM HEPES, 0.5% DMSO, 38 nM [^3^H]digoxin or [^3^H]rosuvastatin, and 1.75 μM [^14^C]mannitol) was added to the basolateral chamber, and 0.3 mL of blank buffer (i.e. no drug) was added to the apical chamber. The cells were then incubated for various times up to 60 min. For inhibition studies, 500 μM unlabelled TCMCB07 was added to buffer containing radiolabelled digoxin or rosuvastatin. At the designated times, 15 μL of apical buffer and 50 μL of basolateral buffer were sampled. Once all the samples were collected, 0.5 mL of ice‐cold HBSS buffer was added to the well to stop transport after which the cells were rinsed three times with 0.5 mL of ice‐cold HBSS. The filters with monolayers were then detached from the chamber and placed in a scintillation vial, and the cells were lysed with 0.33 mL hyamine hydroxide. Radioactivity and protein measurements were then performed as described previously.

Cellular uptake and transepithelial transport were calculated as described previously by our laboratory.^16^, ^17^, ^18^, ^19^ [^3^H]mannitol or [^14^C]mannitol was used to correct the uptake of [^14^C]substrate or [^3^H]substrate, respectively, due to filter binding and extracellular content, as well as the transepithelial transport of substrate due to paracellular flux.

The HEK293‐transfected and MDCKII‐transfected cell systems used in our studies were all obtained from other sources in which the expression of transfected transporters was already established. Specifically, the OATP1A2,^12^ OATP1B1,^20^ OATP1B3,^20^ OATP2B1,^20^ MDR1,^21^ and BCRP^21^ transporters were validated by real‐time reverse transcription polymerase chain reaction, immunoblots, and functional activity, whereas the OCT2,^10^ OAT1,^11^ OAT3,^11^ MATE1,^10^ and MATE2‐K^10^ transporters were validated by real‐time reverse transcription polymerase chain reaction and functional activity. Using immunofluorescence analysis, OATP1A2 was localized to the plasma membrane of HEK293/OATP1A2 cells.^12^

### Mass balance studies

Twenty‐four hour urinary and faecal recovery experiments were performed for TCMCB07 as described previously by our laboratory.^22^ In brief, mice were fasted the night prior to experimentation for 16–18 h, and then, 100 μL (10 μCi) per 20 g mouse of [^3^H]inulin was administered into the tail vein by intravenous bolus injection. This was followed immediately by dosing 200 μL (5 μCi) per 20 g mouse of [^14^C]TCMCB07 (23.3 mg/kg) by oral gavage. In order to assess the systemic disposition of TCMCB07 in the absence of absorption concerns, intravenous bolus injections of [^3^H]inulin and [^14^C]TCMCB07 were given simultaneously to another group of mice. Each mouse was then placed in a metabolic cage for 24 h, along with food and water, and the urine and faeces were collected. The metabolic cage was washed several times, after which 100 μL aliquots of diluted urine and faeces was placed in separate vials. Radioactivity measurements were then performed as described previously. All animal procedures were approved by the Institutional Animal Care and Use Committee at the University of Michigan, Ann Arbor.

### Data analysis

The inhibitory potential of TCMCB07 was evaluated as^23^:

(1) $$\%\text{Control}=100\cdot \left(1-\frac{I^{n}}{{{\mathrm{IC}}_{50}}^{n}+I^{n}}\right),\;$$

where *% Control* relates to the substrate and IC_50_, *I*, and *n* relate to the inhibitor TCMCB07. IC_50_ is the concentration of inhibitor (*I*) that reduces substrate transport by 50%, and *n* is the slope factor.

The uptake kinetics of TCMCB07 was evaluated in transfected cells by a combination of Michaelis–Menten and non‐saturable components^21^:

(2) $$v=\frac{V_{\max}\cdot C}{K_{m}+C}+K_{d}\cdot C,\;$$

where *v* is the uptake rate, *V*_max_ is the maximum uptake rate, *K*_*m*_ is the Michaelis constant, *K*_*d*_ is the non‐saturable rate constant, and *C* is the substrate concentration of TCMCB07. Uptake kinetics in mock cells was evaluated by a linear term:

(3) v=Slope·C+Yint,

where *Slope* is the non‐saturable rate constant and *Yint* is the *y*‐intercept of the line.

The permeability of digoxin and rosuvastatin (prototypical substrates for MDR1 and BCRP, respectively) was determined by the following^18^:

(4) $$P_{\mathrm{app}}=\frac{C_{R}\cdot V_{R}}{{C_{D}}^{o}\cdot A\cdot ∆\Delta t}\;,\;$$

where *P*_*app*_ is the apparent permeability, *C*_*R*_ is the concentration of substrate in the receiver chamber, *C*_*D*_^*o*^ is the concentration of substrate in the donor chamber at time zero, *V*_*R*_ is the volume of buffer in the receiver chamber, *A* is the surface area of the insert filter membrane, and *∆t* is the time over which permeability is measured. The efflux ratio of substrate, in the absence and presence of TCMCB07, was calculated as^24^

(5) $$\mathrm{ER}=\frac{P_{\mathrm{app}}\;\left(B\;\text{to}\;A\right)}{P_{\mathrm{app}}\;\left(A\;\text{to}\;B\right)},\;$$

where *P*_*app*_ (*B to A*) is the permeability of substrate from the basolateral to apical chambers and *P*_*app*_ (*A to B*) is the permeability of substrate from the apical to basolateral chambers. In the absence of TCMCB07, the efflux ratio was referenced to digoxin (*ER*_*Dig*_) or rosuvastatin (*ER*_*Ros*_). In the presence of TCMCB07, the efflux ratio was referenced to digoxin (*ER*_*Dig*+*T*_) or rosuvastatin (*ER*_*Ros*+*T*_). The % change in digoxin *ER*, due to TCMCB07, can then be quantified as:

(6) $$\%\text{Change}\;\mathrm{ER}=\frac{100\cdot \left({\mathrm{ER}}_{\mathrm{Di}g}-{\mathrm{ER}}_{\mathrm{Dig}+T}\right)}{{\mathrm{ER}}_{\mathrm{Dig}}}=100\cdot \left(1-\frac{{\mathrm{ER}}_{\mathrm{Dig}+T}}{{\mathrm{ER}}_{\mathrm{Dig}}}\right).\;$$

A similar analysis can be performed for rosuvastatin by substituting *ER*_*Ros*_ for *ER*_*Dig*_ and *ER*_*Ros*+*T*_ for *ER*_*Dig*+*T*_ in Eqn 6.

Data were reported as mean ± standard error unless otherwise indicated. Non‐linear and linear regression analyses were performed using Prism v7.0 (GraphPad Software, Inc., La Jolla, CA) and a weighting factor of unity or 1/y. Quality of the fit was evaluated by standard error of the parameters, the coefficient of determination (*r* ^2^), and by visual inspection of the residuals. Statistical differences were evaluated using a two‐tailed Student's *t*‐test. A *P* value of ≤0.05 was considered significant.

## Results

The development of TCMCB07 was the result of searching the literature for reports of peptides with unexplained transepithelial transport properties.^7^ Structural comparisons showed that many of these peptides had similarities, effectively producing a transport peptide library. These peptides also exhibited hepatic transport from blood to bile via a multispecific bile acid transporter,^25^ as well as kidney and BBB transport.^26^, ^27^ This class of compounds included synthetic analogues of somatostatin,^25^ opioids,^28^, ^29^ endothelins,^30^, ^31^ renin inhibitors,^32^, ^33^ and natural peptides from fungi and plants.^34^ TCMCB07 was the product of refining structural features common to this class.^7^ Subsequently, cationic peptide transport was discovered in members of the OATP and OAT families of solute transporters, including those with multispecific bile transport capabilities.^8^, ^35^, ^36^, ^37^ Several of these transporters are located in the liver, intestine, kidney, and BBB (blood to brain direction) and are therefore potential candidates for TCMCB07 transport.

### Cell culture studies in HEK293/OATP1B1, HEK293/OATP1B3, HEK293/OATP2B1, HEK293/OATP1A2, HEK293/OAT1, HEK293/OAT3, HEK293/OCT2, HEK293/MATE1, and HEK293/MATE2‐K

Preliminary studies were performed to determine the uptake vs. time profiles of prototypical substrates in mock and transfected cells and the best time for studying TCMCB07 inhibition under linear uptake conditions. Transfected cell studies demonstrated substantially greater uptake of substrate than those in mock cells in which the mean enhancement (i.e. transfected/mock) was 43.4 for estradiol‐17‐β‐d‐glucuronide at 1.5 min (OATP1B1), 4.7 for cholecystokinin octapeptide at 5.0 min (OATP1B3), 63.5 for estrone 3‐sulfate (E3S) at 1.5 min (OATP2B1), 46.1 for E3S at 1.0 min (OATP1A2), 2196 for p‐aminohippurate at 5.0 min (OAT1), 470 for E3S at 5.0 min (OAT3), 373 for 1‐methyl‐4‐phenylpyridinium at 5.0 min (OCT2), 122 for tetraethylammonium at 2.0 min (MATE1), and 46.7 for tetraethylammonium at 2.0 min (MATE2‐K).

**Figure 2:**
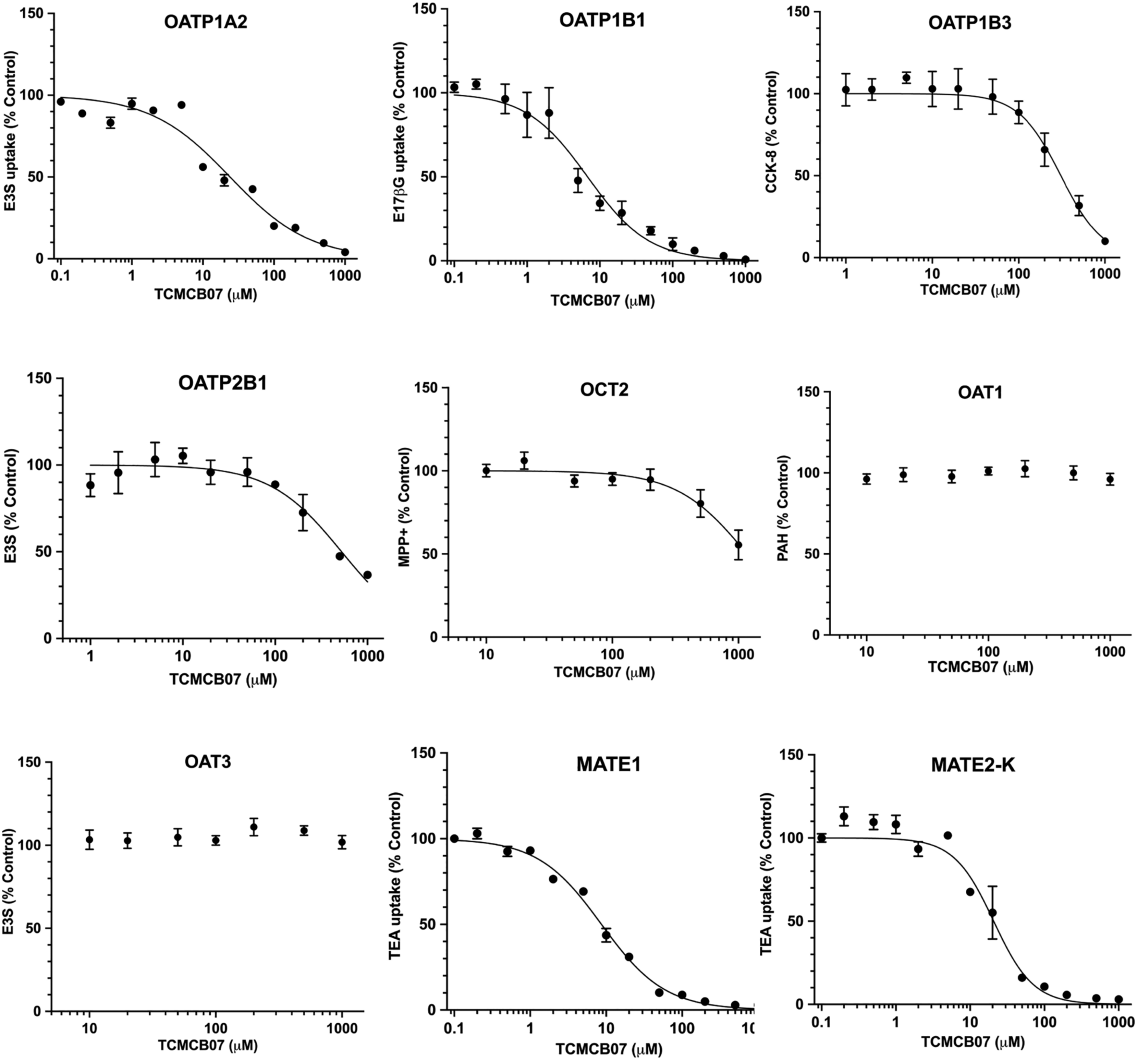
Dose–response curves showing the inhibition of prototypical substrate uptake by TCMCB07 in human transporter‐transfected cells. Incubation conditions were 1.0 μM [^3^H]E3S at 1.0 min for OATP1A2, 1.0 μM [^3^H]E17βG at 1.5 min for OATP1B1, 0.1 μM [^3^H]CCK‐8 at 5 min for OATP1B3, 1.0 μM [^3^H]E3S at 1.5 min for OATP2B1, 1.0 μM [^3^H]MPP^+^ at 5 min for OCT2, 1.0 μM [^14^C]PAH at 5.0 min for OAT1, 1.0 μM [^3^H]E3S at 5.0 min for OAT3, and 1.0 μM [^14^C]TEA at 2.0 min for MATE1 and MATE‐2 K. Unlabelled TCMCB07 was co‐incubated over the 0.1–1000 μM concentration range. See *Table* 1 for IC_50_ values and abbreviations.

Inhibition studies were then performed in transfected cell lines to test whether or not TCMCB07 would inhibit the uptake of substrates in these select transporters. As shown in *Figure* *2*, TCMCB07 inhibited substrate uptake in a dose‐dependent manner in cell cultures transfected with OATP1A2, OATP1B1, OATP1B3, OATP2B1, OCT2, MATE1, and MATE2‐K but not in cell cultures transfected with OAT1 or OAT3. On the basis of IC_50_ values (*Table* *1*), it appears that the inhibitory potency of TCMCB07 is greater for OATP1B1 (IC_50_ 6.8 μM) > MATE1 (IC_50_ 8.7 μM) > MATE2‐K (IC_50_ 20.7 μM) > OATP1A2 (IC_50_ 24.0 μM) > OATP1B3 (IC_50_ 307 μM) > OATP2B1 (IC_50_ 524 μM) > OCT2 (IC_50_ 1,169 μM). IC_50_ values could not be determined for p‐aminohippurate in OAT1 and E3S for OAT3 over the inhibitor concentrations tested (i.e. up to 1000 μM TCMCB07).

**Table 1:** Inhibition of prototypical substrates by TCMCB07 in HEK293 cells transfected with human transporters

Transporter	Gene	Substrate	IC_50_ (μM)	Slope factor	*r* ^2^
OATP1A2	*SLCO1A2*	E3S	24.0 ± 2.8	0.8 ± 0.1	0.945
OATP1B1	*SLCO1B1*	E17βG	6.8 ± 1.0	1.0 ± 0.1	0.923
OATP1B3	*SLCO1B3*	CCK‐8	307 ± 39	1.8 ± 0.3	0.870
OATP2B1	*SLCO2B1*	E3S	524 ± 83	1.1 ± 0.2	0.781
OCT2	*SLC22A2*	MPP^+^	1,169 ± 200	1.6 ± 0.4	0.709
OAT1^a^	*SLC22A6*	PAH	—	—	—
OAT3^a^	*SLC22A8*	E3S	—	—	—
MATE1	*SLC47A1*	TEA	8.7 ± 0.4	1.0 ± 0.1	0.989
MATE2‐K	*SLC47A2*	TEA	20.7 ± 2.1	1.6 ± 0.2	0.949

CCK‐8, cholecystokinin octapeptide; E17βG, estradiol‐17‐β‐d‐glucuronide; E3S, estrone 3‐sulfate; MPP^+^, 1‐methyl‐4‐phenylpyridinium; PAH, p‐aminohippurate; TEA, tetraethylammonium.

Inhibition was evaluated by Eqn 1: %*Control* = 100 · [1 − (*I*^*n*^/(IC_50_^*n*^+*I*^*n*^))], where *% Control* relates to the substrate and IC_50_, *I* and *n* relate to the inhibitor TCMCB07. IC_50_ is the concentration of inhibitor (*I*) that reduces substrate transport by 50%, and *n* is the slope factor. *r* ^2^ is the coefficient of determination. Substrates were given as either [^3^H]radiolabel or [^14^C]radiolabel, along with unlabelled TCMCB07. Data reported as mean ± standard error (*n* = 3 with each experiment run in triplicate).

^a^ OAT1 and OAT3 were not inhibited by PAH and E3S, respectively, over the inhibitory concentration studied for TCMCB07.

### Cell culture studies in MDCKII/MDR1 and MDCKII/BCRP

Preliminary studies were performed in mock and transfected cells in order to determine the permeability of digoxin, a prototypical substrate of MDR1, in both the basolateral to apical (B to A) and apical to basolateral (A to B) directions. For MDCKII mock cells, the mean permeability of digoxin was 20.8 × 10^−6^ cm/s (B to A) and 6.3 × 10^−6^ cm/s (A to B), and in MDR1‐transfected cells, the mean permeability was 22.4 × 10^−6^ cm/s (B to A) and 1.1 × 10^−6^ cm/s (A to B). As a result, the mean efflux ratio (as defined in Eqn 5) was 24.2 in MDR1‐transfected cells as compared with 4.5 in MDCKII mock cells, indicating an enhancement (i.e. transfected/mock) of 4.4 (*P* = 0.0199, as compared with 1.0, indicating no enhancement). As shown in *Table* *2*, in the presence of TCMCB07, the MDR1‐mediated permeability of digoxin was increased by 68% in the B to A direction and decreased by 27% in the A to B direction. Thus, the efflux ratio of digoxin was increased from 24.2 to 51.9 by TCMCB07, with a change in efflux ratio of −152% (*P* = 0.0855). This is opposite to what one would expect from an inhibitor of MDR1, suggesting instead that TCMCB07 was inhibiting one or several influx transporters for digoxin.

**Table 2:** Permeability, efflux ratio, and the % change efflux ratio of digoxin or rosuvastatin (±500 μM TCMCB07) in cell cultures transfected with either human P‐gp (*ABCB1*, *MDR1*) or human BCRP (*ABCG2*)

MDCKII/MDR1	Digoxin	*Dig* + *T*	MDCKII/BCRP	Rosuvastatin	*Ros* + *T*
*P*_*app*_ (*B to A*) ^a^	22.4 ± 4.0	37.6 ± 2.1^*^	*P*_*app*_ (*B to A*)	76.8 ± 4.1	105 ± 2^**^
*P*_*app*_ (*A to B*) ^a^	1.1 ± 0.3	0.8 ± 0.1	*P*_*app*_ (*A to B*)	11.0 ± 0.1	11.5 ± 0.2
Efflux ratio, ${\mathrm{ER}}_{\mathrm{Dig}\pm T}\;\frac{P_{\mathrm{app}}\;\left(B\;\text{to}\;A\right)}{P_{\mathrm{app}}\;\left(A\;\text{to}\;B\right)\;}$	24.2 ± 7.3	51.9 ± 10.8	Efflux ratio, ${\mathrm{ER}}_{\mathrm{Ros}\pm T}\;\frac{P_{\mathrm{app}}\;\left(B\;\text{to}\;A\right)}{P_{\mathrm{app}}\;\left(A\;\text{to}\;B\right)\;}$	7.0 ± 0.3	9.1 ± 0.1^**^
*% Change ER* $100\cdot \left(1-\frac{{\mathrm{ER}}_{\mathrm{Dig}+T}}{{\mathrm{ER}}_{\mathrm{Dig}}}\right)$	0	−152 ± 67	*% Change ER* $100\cdot \left(1-\frac{{\mathrm{ER}}_{\mathrm{Ros}+T}}{{\mathrm{ER}}_{\mathrm{Ros}}}\right)$	0	−32 ± 7^*^

Permeability, efflux ratio (*ER*), and the *% change ER* were calculated according to Eqns 4, 5, and 6, respectively. Digoxin and rosuvastatin were given as [^3^H]radiolabel, and TCMCB07 was unlabelled. Data reported as mean ± standard error (*n* = 3 with each experiment run in triplicate). *Dig* + *T* were studies with digoxin in the presence of TCMCB07; *Ros* + *T* were studies with rosuvastatin in the presence of TCMCB07.

^a^ Permeability (*P*_*app*_) values were multiplied ×10^6^ and shown in units of cm/s.

^*^ *P* < 0.05 for *Dig* + *T* compared with digoxin alone, and for *Ros + T* compared with rosuvastatin alone, as determined by a two‐tailed Student's *t*‐test

^**^ *P* < 0.01 for *Ros* + *T* compared with rosuvastatin alone, as determined by a two‐tailed Student's *t*‐test.

Similar studies were performed in mock and transfected cells with rosuvastatin, a prototypical substrate of BCRP. For MDCKII mock cells, the mean permeability of rosuvastatin was 55.0 × 10^−6^ cm/s (B to A) and 18.0 × 10^−6^ cm/s (A to B), and in BCRP‐transfected cells, the mean permeability was 76.8 × 10^−6^ cm/s (B to A) and 11.0 × 10^−6^ cm/s (A to B). As a result, the mean efflux ratio (as defined in Eqn 5) was 7.0 in MDR1‐transfected cells as compared with 3.1 in MDCKII mock cells, indicating an enhancement (i.e. transfected/mock) of 2.3 (*P* = 0.0001, as compared with 1.0, indicating no enhancement). As also shown in *Table* *2*, in the presence of TCMCB07, the BCRP‐mediated permeability of rosuvastatin was increased by 37% in the B to A direction and by 5% in the A to B direction. Thus, the efflux ratio of rosuvastatin was only increased from 7.0 to 9.1 by TCMCB07, with a minimal change in efflux ratio of only −32% (*P* = 0.0101). This change does not support a role for TCMCB07 in BCRP inhibition, nor is the directional change strong enough (i.e. ≥50%) to suggest that TCMCB07 is an inhibitor of influx transporters for rosuvastatin.

### Cell culture studies investigating the uptake of [^14^C]TCMCB07

**Figure 3:**
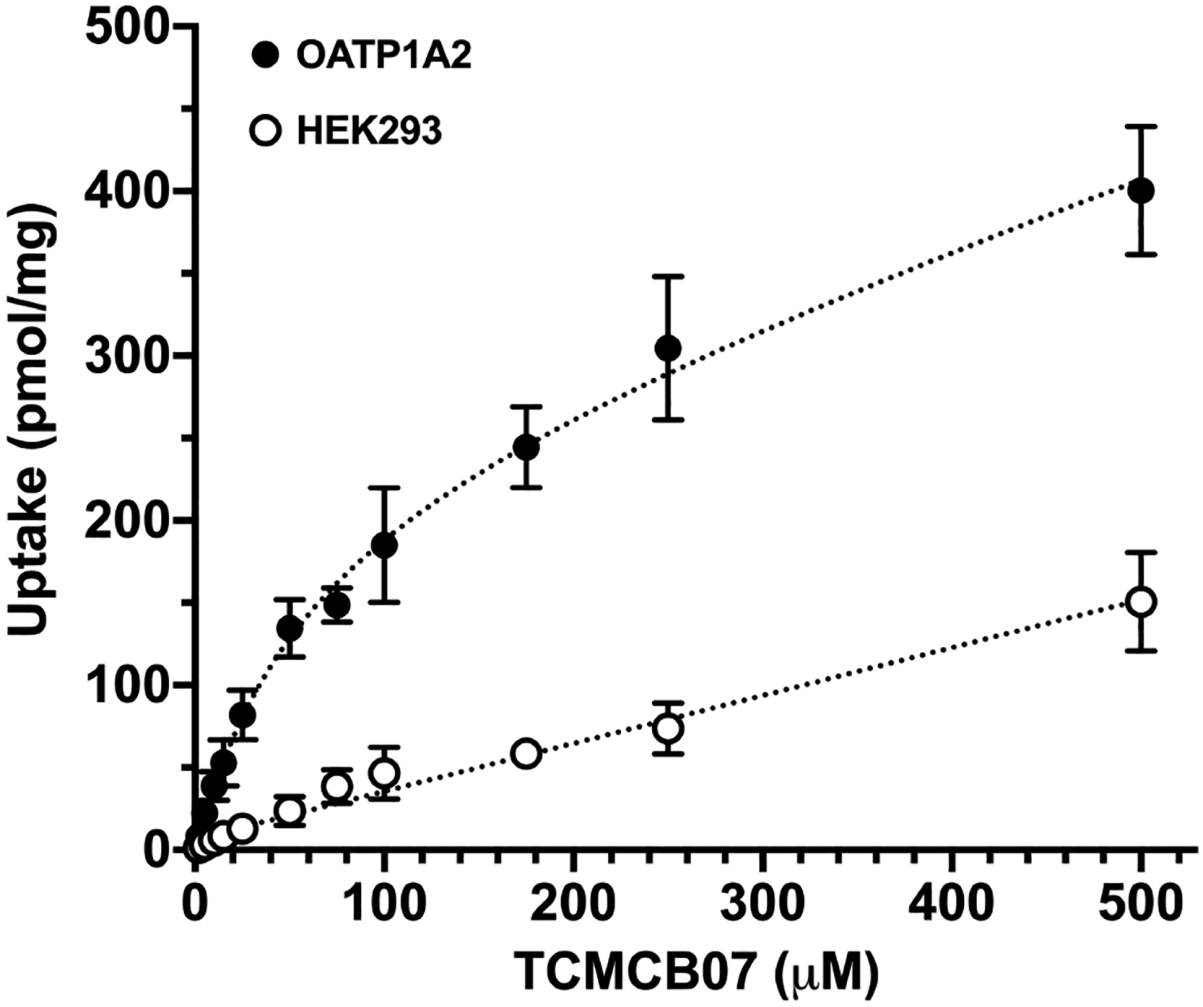
Concentration‐dependent uptake of [^14^C]TCMCB07 in HEK293 mock (open circles) and human OATP1A2‐transfected (closed circles) cells. See *Table* 3 for the saturable and non‐saturable (linear) uptake parameters.

TCMCB07 was shown to inhibit the uptake of prototypical substrates in HEK293/OATP1B1, HEK293/OATP1B3, HEK293/OATP2B1, HEK293/OATP1A2, HEK293/OCT2, HEK293/MATE1, and HEK293/MATE2‐K, suggesting indirectly that this cyclic nonapeptide may also be a substrate for these transporters. As a result, [^14^C]TCMCB07 was synthesized to directly test whether or not this compound was also a substrate. Initial studies showed very little, if any, uptake over 10 min for TCMCB07 in OATP1B1, OATP1B3, OATP2B1, OCT2, MATE1, and MATE2‐K cell cultures, where transfected over mock ratios were ≤1.6. However, for OATP1A2, the uptake of TCMCB07 was 3–4 times greater at 5 min as compared with mock cells. Thus, the uptake of TCMCB07 was studied further in HEK293/OATP1A2 cells to evaluate if this compound displayed saturable (i.e. capacity‐limited) kinetics. As shown in *Figure* *3*, the uptake of TCMCB07 was non‐linear in OATP1A2‐transfected cells but linear in HEK293 mock cells. According to the shape of the curve and after testing various regression models, the data were best fit to a single Langmuir plus linear term for OAT1A2‐transfected cells and to a linear term for mock cells (*Table* *3*). The non‐saturable rate constant (*K*_*d*_) in transfected cells was similar to that determined in mock cells (*Slope*). Moreover, at low concentrations (i.e. << *K*_*m*_), the saturable uptake component (*V*_max_/*K*_*m*_) accounted for over 90% of the total uptake (*V*_max_/*K*_*m*_ + *K*_*d*_) of TCMCB07 in OATP1A2‐transfected cells.

**Table 3:** Transport kinetics for the cellular uptake of TCMCB07 in HEK293 mock and human OATP1A2‐transfected cells

Cells	*V*_max_ (pmol/mg)	*K*_*m*_ (μM)	*K*_*d*_ (μL/mg)	*Slope* (μL/mg)	*Yint* (pmol/mg)	*r* ^2^
Mock				0.29 ± 0.01	6.47 ± 2.35	0.983
Transfected	236 ± 63	58.4 ± 22.0	0.39 ± 0.14			0.946

Uptake kinetics in transfected cells (at 5 min) was evaluated by a combination of Michaelis–Menten and non‐saturable components such that $v=\left[V\max\cdot \frac{C}{\mathrm{Km}+C}+\mathrm{Kd}\cdot C\right],$ where *v* is the uptake rate, *V*_max_ is the maximum uptake rate, *K*_*m*_ is the Michaelis constant, *K*_*d*_ in the non‐saturable rate constant, and *C* is the substrate concentration of TCMCB07. Uptake kinetics in mock cells (at 5 min) was evaluated by a linear term, such that *v* = [*Slope* · *C*+*Yint*], where *Slope* is the non‐saturable rate constant and *Yint* is the *y*‐intercept of the line. *r* ^2^ is the coefficient of determination. TCMCB07 was given as [^14^C]radiolabel. Data reported as mean ± standard error (*n* = 3, with each experiment run in triplicate).

### Mass balance studies

Since TCMCB07 was a substrate for OATP1A2, which is localized to the small intestine,^38^ these studies were initiated to examine whether or not TCMCB07 was orally absorbed under *in vivo* conditions. As shown in *Table* *4*, TCMCB07 was eliminated primarily in the bile and recovered in the faeces after both routes of administration (i.e. 67.6/(3.8 + 67.6) = 95% for oral and 49.4/(15.8 + 49.4) = 76% for intravenous). In contrast, complete urinary recovery of intravenously administered inulin was observed when given together with oral or intravenous TCMCB07, indicating that urine was not lost during the collection process. The oral absorption of TCMCB07, estimated by comparing the urinary recovery of peptide after oral and intravenous dosing, was 24.2% (*P* = 0.0033, as compared with 0, indicating no absorption). Assuming that 76% of the faecal excretion was biliary (after intestinal absorption), the oral absorption of TCMCB07 may be as high as 55% [calculated as (0.76 × 67.6) + 4].

**Table 4:** *In vivo* mass balance study in mice following both oral and intravenous administrations of 23.3 mg/kg TCMCB07

Mouse number	Oral TCMCB07	Oral TCMCB07	Intravenous inulin	Mouse number	Intravenous TCMCB07	Intravenous TCMCB07	Intravenous Inulin
	% Urinary recovery	% Faecal recovery	% Urinary recovery		% Urinary recovery	% Faecal recovery	% Urinary recovery
1	5.0	52.4	106	1	14.0	53.8	104
2^a^	4.8	71.7	100	2	20.1	29.7	93.2
3	2.9	74.0	107	3	13.4	64.7	95.1
4	2.6	72.1	107				
							
Mean	3.8	67.6	105	Mean	15.8^*^	49.4	97.4
SE	0.6	4.4	1.3	SE	1.7	8.4	2.7

SE, standard error.

Mice were injected with [^3^H]inulin by intravenous bolus injection and then dosed with [^14^C]TCMCB07 by oral gavage. [^3^H]inulin and [^14^C]TCMCB07 were also given simultaneously by intravenous bolus injection to another group of mice. Each mouse was then placed in a metabolic cage, and the urine and faeces were collected over 24 h.

^a^ Urine and faeces were collected over 48 h.

^*^ *P* < 0.01 compared with % urinary recovery of oral TCMCB07, as determined by a two‐tailed Student's *t*‐test. No statistical differences were observed between oral and intravenous TCMCB07 in % faecal recovery and % urinary recovery of intravenous inulin when co‐administered with either oral or intravenous TCMCB07.

## Discussion

Cachexia is among the most incapacitating and life‐threatening conditions, especially in cancer. Although the mechanisms underlying cachexia are complex, the pathology of this condition includes a reduced food intake accompanied by increased energy expenditure, leading to increased catabolism and muscle wasting. Although several pharmacological approaches have been tested, a particularly attractive strategy involves blocking the MC4R. In this regard, a significant effort is underway to identify selective, potent, safe, and orally active MC4R antagonists.^39^, ^40^ More recently, the cyclic nonapeptide TCMCB07, an MC4R antagonist, was shown to stimulate food intake and weight gain in rats after 10 mg/kg oral dosing.^7^ This finding suggests that TCMCB07 traverses the epithelial cells of the small intestine for drug absorption as well as the endothelial cells of the BBB to exert its pharmacologic effect. However, to further advance its preclinical development and study of TCMCB07 in human patients, it is critical to understand the mechanisms by which this compound is transported across biological membranes such as the intestine, liver, and brain.

According to Food and Drug Administration (FDA) guidelines,^41^ an investigational drug should be studied *in vitro* in cell culture systems expressing the transporters OATP1B1, OATP1B3, OAT1, OAT3, OCT2, MATE1, MATE2‐K, P‐gp (MDR1), and BCRP. Along with these nine transporters, we further studied TCMCB07 in OATP2B1‐transfected and OATP1A2‐transfected cells. Thus, TCMCB07 was evaluated for its inhibitory potential of prototypical substrates for these transporters and its potential not only as an inhibitor but also as a substrate for these transporters. Finally, mass balance studies were performed in mice to validate the *in vivo* oral absorption of TCMCB07, as indicated by the *in vitro* studies conducted in cell culture. Specifically, several major findings were revealed: (i) TCMCB07 was an inhibitor of the solute carrier transporters OATP1A2, OATP1B1, OATP1B3, OATP2B1, OCT2, MATE1, and MATE2‐K; (ii) TCMCB07 was a substrate for OATP1A2 but not for the transporters OATP1B1, OATP1B3, OATP2B1, OCT2, MATE1, and MATE2‐K; (iii) TCMCB07 exhibited a combination of Michaelis–Menten (*V*_max_ = 236 pmol/mg; *K*_*m*_ = 58.4 μM) and non‐saturable (*K*_*d*_ = 0.39 μL/mg) transport kinetics in OATP1A2‐transfected cells where, at concentrations << *K*_*m*_, the saturable component accounted for over 90% of its total uptake; (iv) TCMCB07 was eliminated primarily in the bile and recovered in the faeces after both oral and intravenous administrations in mice; and (v) oral absorption of TCMCB07 in mice was calculated at 24.2% but may be as high as 55%.

According to the HUGO Gene Nomenclature Committee database, there are currently 423 solute carrier^42^ and 51 adenosine triphosphate‐binding cassette transporters^43^ genes that have been cloned from humans. The transporters proposed for evaluation during drug development are considerably less, as reported previously by the FDA^41^ and others.^8^, ^44^ ^.^Interestingly, TCMCB07 was found not to be a substrate for any of the nine transporters recommended by the FDA, although it did inhibit several of them. However, given the fact that TCMCB07 was of a larger size (i.e. nonapeptide) and substantially excreted into bile, we considered other candidate transporters. In general, OATPs including OATP1A2 mediate the uptake of bile acids into intestinal epithelia and hepatocytes.^38^, ^45^, ^46^ Some members of the OATP family have also been reported to transport larger peptides such as the synthetic opioid pentapeptide DADLE and heptapeptide deltorphin II.^47^ In particular, the cyclic pentapeptide DPDPE was shown to be transported by OATP‐A, OATP‐C, and OATP‐8 (currently named OATP1A2, OATP1B1, and OATP1B3, respectively). In our studies, we found that while OATP1B1 and OATP1B3 were inhibited by TCMCB07, the uptake of [^14^C]TCMCB07 in transfected cell cultures was minimal, at best, with transporter/mock ratios of only 1.5. However, [^14^C]TCMCB07 was found to be both an inhibitor of and a substrate for OATP1A2.

**Figure 4:**
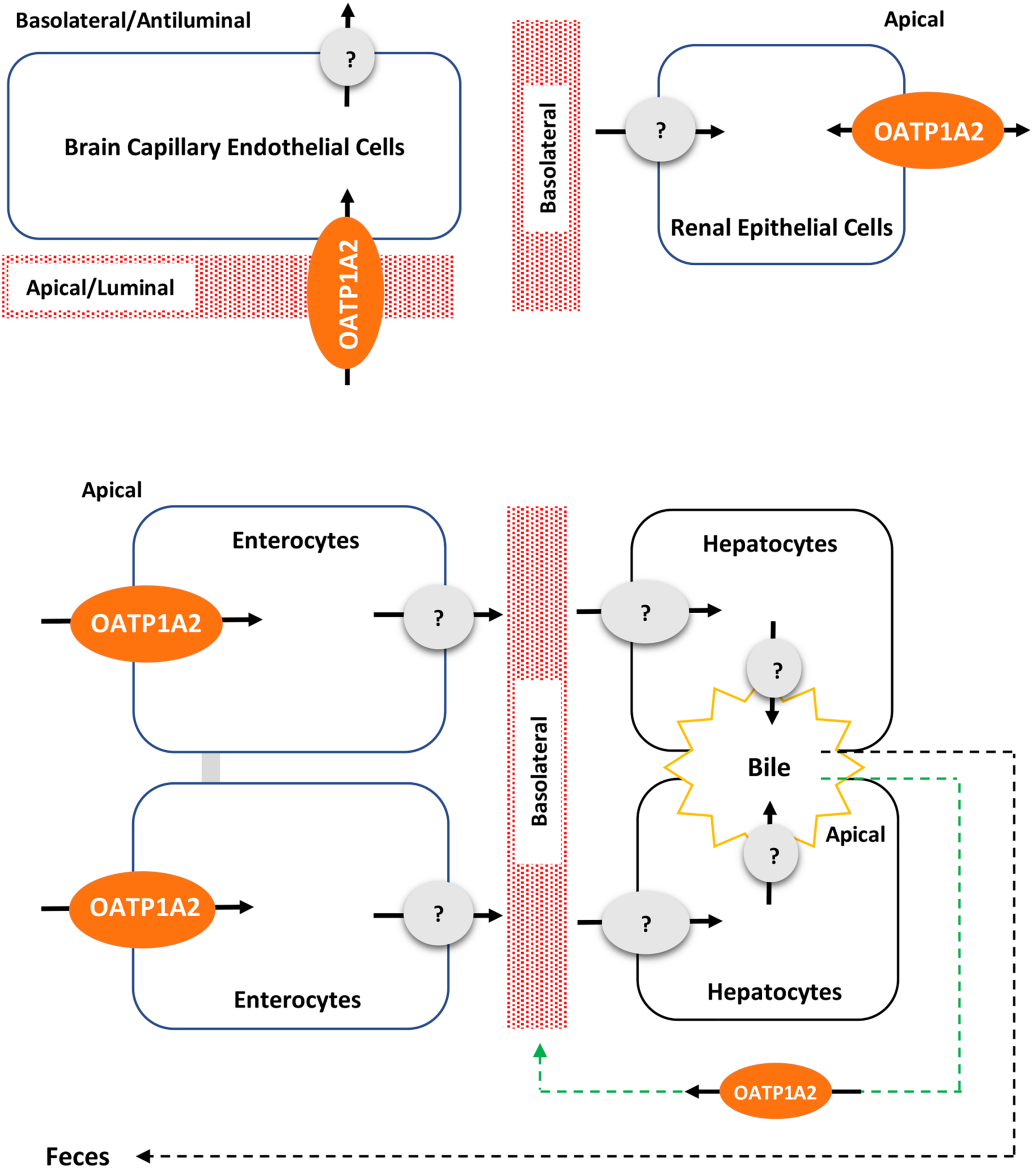
Schematic drawing for the human OATP1A2‐mediated transport of TCMCB07 across the biological membranes of the intestine, liver, kidney, and brain. Cellular uptake of TCMCB07 occurs at the apical membrane of enterocytes in the intestine, the cholangiocytes in the liver, and the apical/luminal membrane of capillary endothelial cells in the brain; TCMCB07 is most likely secreted (effluxed) at the apical membrane of distal epithelial cells in the kidney, although reabsorption into the cell is also possible. The green arrow represents the biliary uptake of TCMCB07 into cholangiocytes where it is reabsorbed back into the blood, the black arrow represents the biliary excretion of TCMCB07 into faeces, and the red rectangular patterns represent the blood.

*Figure* *4* is based on the reported localization of OATP1A2 in the intestine, liver, kidney, and brain,^36^, ^44^, ^48^, ^49^ where the transporter can explain the absorption, disposition, and pharmacologic response of TCMCB07. Thus, TCMCB07 is absorbed across the apical membranes of intestinal epithelia by OATP1A2; however, it is unclear how the nonapeptide is effluxed from the enterocyte into blood. It is also unclear how TCMCB07 is taken up into hepatocytes and excreted into bile. Although OATP1B1, OATP1B3, and OATP2B1 provide a mechanism for sinusoidal uptake, TCMCB07 is not a substrate for these transporters. Likewise, TCMCB07 is not a substrate for MATE1 and MATE2‐K, which are responsible for drug efflux across the canalicular membranes and excretion into bile. OATP1A2 is expressed in the liver but not in hepatocytes. Instead, OATP1A2 is expressed in cholangiocytes lining the bile ducts where it can return TCMCB07 to liver sinusoids via the cholehepatic shunt. In the kidney, OCT2 is the first step for renal tubular secretion where drug is transported across the basolateral membrane of epithelial cells. Although TCMCB07 could inhibit this transporter, the nonapeptide is not a substrate for OCT2. Once in the cell, it is likely that TCMCB07 is effluxed into the urine by OATP1A2, although this transporter may also be involved in reabsorption. Finally, OATP1A2 is expressed in endothelial cells of the BBB, thereby allowing TCMCB07 to exert its pharmacologic response.

Mass balance studies clearly demonstrate that based on 24 h urinary recoveries, 24.2% of TCMCB07 is absorbed after oral dosing. Given the extensive biliary excretion of TCMCB07 after intravenous dosing (i.e. 76% of total recovery), it is very likely that the faecal excretion of TCMCB07 after oral dosing represents both unabsorbed drug and drug that was absorbed, subsequently excreted into the bile and then collected in the faeces. Taking this into account, we estimate that as much as 55% of orally administered TCMCB07 may be absorbed in the intestine.

In conclusion, OATP1A2 is responsible for the transporter‐mediated uptake of TCMCB07 in the intestine and brain, thereby allowing the drug to be administered by the oral route and exhibit anti‐cachexia activity. Future studies will have to translate these *in vitro* studies in transfected cell systems, and *in vivo* studies in mice, to humans by demonstrating efficacy and safety in patient populations.

## Conflict of interest

Y.H. and D.E.S declared no conflict of interest. K.A.G is a chief scientific officer and a principal shareholder of Tensive Controls Inc. and is the inventor on the patent for TCMCB07.

## Funding

This work was supported in part by the National Institutes of Health National Cancer Institute (grant number R44CA210763).
